# Adrenal cortex expression quantitative trait loci in a German Holstein × Charolais cross

**DOI:** 10.1186/s12863-016-0442-x

**Published:** 2016-10-06

**Authors:** Bodo Brand, Markus O. Scheinhardt, Juliane Friedrich, Daisy Zimmer, Norbert Reinsch, Siriluck Ponsuksili, Manfred Schwerin, Andreas Ziegler

**Affiliations:** 1 Institute for Genome Biology, Leibniz Institute for Farm Animal Biology, Wilhelm-Stahl-Allee, Dummerstorf, Germany; 2 Current affiliation: Institute for Farm Animal Research and Technology, University of Rostock, Justus-von-Liebig-Weg, 18059 Rostock, Germany; 3 Institute of Medical Biometry and Statistics, University of Lübeck, Ratzeburger Allee, Lübeck, Germany; 4 Institute for Farm Animal Research and Technology, University of Rostock, Justus-von-Liebig-Weg, Rostock, Germany; 5 Institute for Genetics and Biometry, Leibniz Institute for Farm Animal Biology, Wilhelm-Stahl-Allee, Dummerstorf, Germany; 6 Center for Clinical Trials, University of Lübeck, Ratzeburger Allee, Lübeck, Germany; 7 School of Mathematics, Statistics and Computer Science, University of KwaZulu-Natal, Pietermaritzburg, South Africa

**Keywords:** Gene expression, Cow, Adrenal cortex, eQTL analysis, Pathway analysis

## Abstract

**Background:**

The importance of the adrenal gland in regard to lactation and reproduction in cattle has been recognized early. Caused by interest in animal welfare and the impact of stress on economically important traits in farm animals the adrenal gland and its function within the stress response is of increasing interest. However, the molecular mechanisms and pathways involved in stress-related effects on economically important traits in farm animals are not fully understood. Gene expression is an important mechanism underlying complex traits, and genetic variants affecting the transcript abundance are thought to influence the manifestation of an expressed phenotype. We therefore investigated the genetic background of adrenocortical gene expression by applying an adaptive linear rank test to identify genome-wide expression quantitative trait loci (eQTL) for adrenal cortex transcripts in cattle.

**Results:**

A total of 10,986 adrenal cortex transcripts and 37,204 single nucleotide polymorphisms (SNPs) were analysed in 145 F2 cows of a Charolais × German Holstein cross. We identified 505 SNPs that were associated with the abundance of 129 transcripts, comprising 482 cis effects and 17 trans effects. These SNPs were located on all chromosomes but X, 16, 24 and 28. Associated genes are mainly involved in molecular and cellular functions comprising free radical scavenging, cellular compromise, cell morphology and lipid metabolism, including genes such as *CYP27A*1 and *LHCGR* that have been shown to affect economically important traits in cattle.

**Conclusions:**

In this study we showed that adrenocortical eQTL affect the expression of genes known to contribute to the phenotypic manifestation in cattle. Furthermore, some of the identified genes and related molecular pathways were previously shown to contribute to the phenotypic variation of behaviour, temperament and growth at the onset of puberty in the same population investigated here. We conclude that eQTL analysis appears to be a useful approach providing insight into the molecular and genetic background of complex traits in cattle and will help to understand molecular networks involved.

**Electronic supplementary material:**

The online version of this article (doi:10.1186/s12863-016-0442-x) contains supplementary material, which is available to authorized users.

## Background

eQTL analyses and the integration of proteomic and metabolomics data in genetic analyses contributed to the understanding of genes and molecular networks of complex traits in humans [1, 2] and farm animals [3–5]. In cattle most studies investigating the genetic background of complex traits such as milk performance or reproduction used quantitative trait loci (QTL) mapping and genome-wide association studies. Recent advances of high throughput technologies promoted the utilization of transcriptomic, proteomic or metabolomics data in genetic analyses [6].

Caused by interest in animal welfare and the impact of stress on economically important traits in farm animals the adrenal gland and its function within the stress response is of increasing interest. Stress-related effects on productivity [7–9], health [10, 11] and reproduction [12, 13] in cattle are not fully understood. However, insight into molecular networks affecting these traits in cattle could contribute to the understanding of the mutual relationship between stress and obesity or stress related effects on health in humans.

Adrenal cortex is a promising target to investigate these effects, due to its impact on stress response and regulation of essential metabolic pathways [14]. Human diseases such as Addison’s disease, the Cushing’s syndrome or Conn’s syndrome that are related to aberrations in glucocorticoid or mineralocorticoid levels further accentuate its relevance [15–17]. In cattle first observations indicating the importance of the adrenal gland for economically important traits were related to reproduction and lactation [18, 19]. Recent studies in cattle and pig showed that genetic variants in the glucocorticoid receptor and mineralocorticoid receptors are associated with cortisol concentrations and meat quality traits, for example [20, 21].

Therefore we investigated the genetic background of adrenocortical gene expression in a F2 cattle population (SEGFAM). The SEGFAM population is a cross of Holstein cows that were selected for high milk yield and Charolais bulls that were selected for meat production for a long time. By crossing the two breeds a high genetic and phenotypic variance in regard to milk and meat production traits was expected due to segregation of different QTL alleles in the F2 cross [22]. The population was established to investigate the genetic and physiological background of nutrient transformation types, and first studies showed a huge phenotypic variance in regard to growth at the onset of puberty [4, 23], milk yield [24] and temperament [25, 26]. By identifying eQTL in this population we assumed to gain insight into the genetic regulation of gene expression of different nutrition types that could provide new targets for further research of genes that have an effect on economically important traits such as milk production and meat quality traits in cattle.

The aims of this study were first to identify eQTL in the adrenal cortex in cattle and secondly, to evaluate whether associated genes and molecular pathways are known to affect economically important traits in cattle. The analysed data comprised genotypes of 37,204 SNPs and expression values for 10,986 transcripts. In order to account for systemic effects and to de-correlate the expression data a sire-dam model was used during pre-processing. To account for relatedness the complete pedigree data comprising the founder and F1 generation was used. Then we performed a genome-wide eQTL analysis using an adaptive linear rank test [27] on the residuals of the sire-dam model and the genotype data. Finally, we compared the results of this study with previous studies within the same population and performed Ingenuity® Pathway Analysis (IPA, QIAGEN Redwood City, www.qiagen.com/ingenuity) and literature reviews to identify the molecular pathways involved and evaluate potential targets for further research.

## Results

### Expression quantitative trait loci

**Fig. 1:**
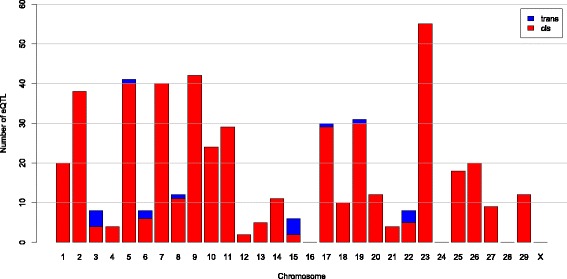
Distribution of eQTL across chromosomes. The number of cis (red) and trans eQTL (blue) per chromosome is shown as bars

**Fig. 2:**
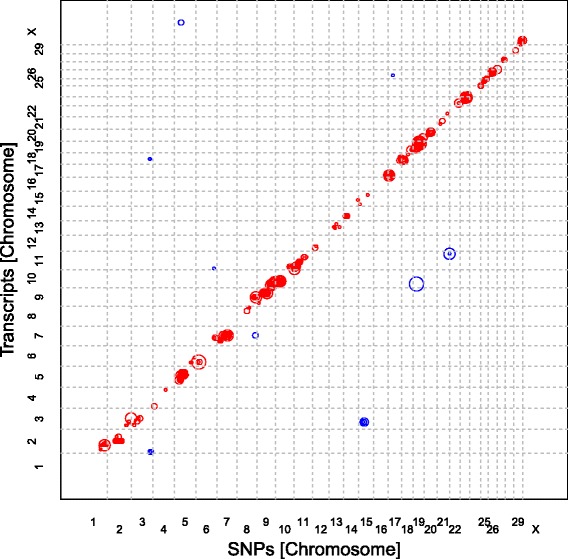
Position of eQTL in regard to the genomic location of the SNP and the gene encoding the transcript. The position of eQTL is plotted in regard to the genomic location of the SNP (x-axis) and the gene encoding the transcripts (y-axis). The size of the circles indicates the magnitude of the *p*-value with larger circles indicating smaller *p*-values. Red circles indicate cis and blue circles trans eQTL

In total, 505 SNPs were identified to be associated with the expression of 129 transcripts. A complete list of eQTL including the chromosomal position and annotation of the SNP and the gene encoding the transcript is provided in Additional file 1. In addition to Benjamini-Hochberg adjusted *p*-values of the adaptive linear rank test we also included the unadjusted *p*-values of adaptive linear rank test, Kruskal Wallis-test and ANOVA for completeness. The number of eQTL per chromosome is shown in Fig. 1. Identified associations and the corresponding genomic position for both SNP and the gene encoding the transcript are shown in Fig. 2. eQTL were identified on all chromosomes except on chromosomes X, 16, 24 and 28. The highest number of associations was observed on chromosome 23 comprising 55 SNPs associated with 11 transcripts. Apparently, in the case of most of these associations (482 of 505) both the SNP and the gene encoding the transcript were located on the same chromosome (cis associations). Only for 17 associations SNPs and genes were located on different chromosomes (trans associations). For the remaining six associations the position of the SNP was unknown.

**Fig. 3:**
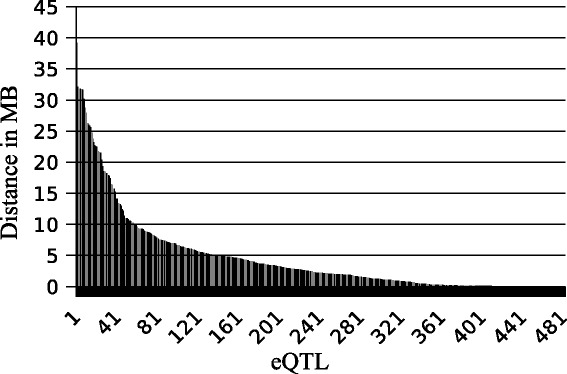
Distance between SNP and the gene encoding the affected transcript. The distance between the genomic location of the SNP and the start position of the gene encoding the transcript is shown for all 482 eQTL for which the SNP and the gene encoding the affected transcript were located on the same chromosome

Genomic distances between SNPs and genes for all 482 cis associations are shown in Fig. 3. Most of these SNPs (343 of 482) were located in close vicinity (less than 5 MB) to the gene encoding the transcript. On the other hand, the expression of *LYPD6B* (Chr. 2), *GRIP1* (Chr. 5), *SNCB* (Chr. 7), *COQ3* (Chr. 9) and *ALDH5A1* (Chr.23), for example, was associated with 21, 15, 35, 28 and 20 SNPs, respectively. These SNPs were located up to 30 MB up- or downstream of the gene encoding the transcript. Considering that we investigated a F2 population that showed considerable linkage disequilibrium ([28] and Additional file 2) a clear discrimination between local and distant or cis and trans eQTL was hindered for these eQTL [29, 30]. Therefore, we assumed a cis or chromosome specific effect for all eQTL for which the SNP and the associated gene were located on the same chromosome. For the 17 eQTL for which the SNP and the gene encoding the transcript were located on different chromosomes a trans-regulatory effect was assumed.

### Ingenuity® pathway analysis

In order to gain insight into molecular and cellular functions and regulatory processes, in which the genes encoding the 129 identified transcripts are involved in, Ingenuity® pathway analysis was performed. The analysis indicated that the genes are mainly involved in molecular and cellular function comprising free radical scavenging, cellular compromise, cell morphology, lipid metabolism and small molecule biochemistry. A complete list of the top five diseases and bio functions including the related genes is provided in Table 1. The results of the Ingenuity® Pathway upstream analyses are provided in Tables 2 and 3. Prolactin was the most important upstream regulator. The most important causal networks were related to *ESRRG*, *GNRH1* and the two retinoic acid receptors *RARG* and *RARB* as master regulators (Table 3). Most of the affected diseases and bio functions as well as the most important causal networks and the most important upstream regulators are related to a relatively small number of genes that are involved in many of the identified pathways and causal networks, such as *CYCS*, *LHCGR*, *TF*, *VIM*, *XDH*, *CYP27A1* and *NQO1*.

**Table 1:** Top five diseases and bio functions of the Ingenuity® pathway analyses

Category	*p*-value	Molecules
Molecular and Cellular Function		
Free Radical Scavenging	2.69E-02-9.18E-02	*TF, EPAS1, NQO1, CYCS, XDH*
Cellular Compromise	2.69E-02-5.98E-02	*VIM, NQO1, CYCS, XDH*
Cell Morphology	2.69E-02-9.04E-02	*VIM, MGAT5, LHCGR, NQO1, CYCS, GDPD2, XDH*
Lipid Metabolism	2.69E-02-9.39E-02	*VIM, EPAS1, NQO1, LHCGR, CYCS, XDH, CYP27A1, PHYH*
Small Molecule Biochemistry	2.69E-02-9.39E-02	*VIM, LYVE1, GSTO1, SLC2A9, EPAS1, NQO1, LHCGR, CYCS, XDH, CYP27A1, PHYH, EXTL2*
Diseases and Disorders		
Inflammatory Response	2.69E-02-9.39E-02	*VIM, GSTO1, TF, MGAT5, EPAS1, NQO1, LHCGR, CYCS, XDH*
Neurological Disease	2.69E-02-9.39E-02	*VIM, GSTO1, TF, MGAT5, LHCGR, NQO1, XDH, CYP27A1, PHYH*
Organismal Injury and Abnormalities	2.69E-02-9.48E-02	*VIM, TF, PTP4A2, MGAT5, EPAS1, LHCGR, NQO1, XDH, PHYH*
Psychological Disorders	2.69E-02-8.3E-02	*VIM, GSTO1, TF, NQO1, CYP27A1, PHYH*
Reproductive System Disease	2.69E-02-8.7E-02	*TF, PTP4A2, LHCGR, PHYH*
Physiological System Development and Function		
Reproductive System Development and Function	2.69E-02-9.39E-02	*TF, PTP4A2, EPAS1, LHCGR*
Tissue Morphology	2.69E-02-9.39E-02	*VIM, TF, EPAS1, LHCGR, NQO1, XDH, PHYH*
Embryonic Development	2.69E-02-7.84E-02	*VIM, LYVE1, TF, PTP4A2, EPAS1, LHCGR, NQO1, GDPD2*
Cardiovascular System Development and Function	2.69E-02-8.7E-02	*VIM, TF, MGAT5, EPAS1, CYCS, XDH*
Hematological System Development and Function	2.69E-02-9.39E-02	*TF, MGAT5, EPAS1, NQO1, LHCGR, CYCS, XDH*

The top five molecular and cellular functions, diseases and disorders as well as physiological system development and functions are displayed, Benjamini-Hochberg adjusted *p*-values and the related genes

**Table 2:** Top five upstream regulators of the Ingenuity® upstream analyses

Upstream regulator	Molecule type	*P*-value of overlap	Target molecules in dataset
PRL	cytokine	6.07E-04	*LHCGR, LYVE1, VIM, XDH*
NRF1	transcription regulator	7.77E-04	*CYCS, NQO1*
MAPK7	kinase	1.13E-03	*NQO1, VIM*
NFE2L2	transcription regulator	2.57E-03	*EPAS1, GSTO1, NQO1*
CHD5	enzyme	2.80E-03	*VIM*

**Table 3:** Top four master regulators of the Ingenuity® upstream analyses

Master regulator	*P*-value of overlap	Target molecules in dataset
ESRRG	5.07E-05	*CYCS, LHCGR, LYVE1, TF, VIM, XDH*
GNRH1	1.44E-04	*CYCS, CYP27A1, EPAS1, EXTL2, LHCGR, LYVE1, NQO1, P2RX5, PHYH, RPL13, SLC2A9, TF, VIM, XDH*
RARG	1.73E-04	*CYCS, CYP27A1, EPAS1, GSTO1, LHCGR, LYVE1, MGAT5, NQO1, TF, VIM, XDH*
RARB	1.81E-04	*CYCS, CYP27A1, EPAS1, GSTO1, LHCGR, LYVE1, MGAT5, NQO1, TF, VIM, XDH*

## Discussion

In order to identify molecular pathways that potentially influence the phenotypic variation of economically important traits in cattle we investigated the genetic background of adrenocortical gene expression in an experimental F2 population of a Charolais × German Holstein cross. We identified 505 SNPs associated with the expression of 129 genes. We also showed that some of these genes are involved in molecular pathways affecting economically important traits in cattle. In comparison with eQTL mapping studies in other species the proportion of cis eQTL (482) compared with trans eQTL (17) observed in our study is high. This is in line with the observation that the number of identified local or cis eQTL is related to sample size, extent of linkage disequilibrium, size of the window applied for cis eQTL and effect size of eQTL which is assumed to be stronger for cis eQTL in comparison with trans eQTL [29–31]. A reason for the high number of cis eQTL observed in our study might be that we assumed a cis or chromosome specific effect for all associations for which SNP and gene were located on the same chromosome. We decided to use this definition for cis associations for two reasons. Firstly, the extent of linkage disequilibrium observed in the studied population is vast ([28] and Additional file 2) and, secondly, the relative genomic position of SNP and associated gene does not necessarily provide information about the underlying regulatory variation [29]. However, even a window size of 1 MB which is often used to differentiate cis and trans effects would result in a higher number of cis eQTL (314) in comparison with trans eQTL (191). In addition, the extent of linkage disequilibrium and the high number of transcripts that are associated with more than one SNP in close vicinity to the gene encoding the transcript also indicated that the identified SNPs are not necessarily the causal variants but are in linkage disequilibrium to the causal polymorphisms and that the identified associations rather indicate genomic regions harbouring the causal variants affecting the transcript abundance.

Ingenuity® pathway analyses of genes encoding affected transcripts revealed that some of the genes are involved in “Diseases and Bio Functions” like lipid metabolism, inflammatory response and reproductive system development and function. This indicates that the identified eQTL potentially contribute to the phenotypic variation of economically important traits in cattle. For some of the genes there is evidence that they have an effect on reproduction, milk performance, growth or health traits, based on genetic and molecular studies in cattle and other farm animals. The following sections comprise details for selected genes. In addition, comparisons with studies within the SEGFAM population indicate that some of the genes affected by eQTL contribute to the phenotypic variation of behaviour, temperament and growth at the onset of puberty. Since the effects of adrenocortical gene expression on most of the economically important traits in cattle still have to be investigated, for other genes only the contribution to disease phenotypes in human or mice or the involvement in specific metabolic pathways like reproductive system development and function suggests that they might contribute to the phenotypic variation of economically important traits in cattle. In addition, the sheer existence of genomic regions affecting the adrenocortical transcript abundance of genes that are known to influence economically important traits in cattle is no evidence that the adrenocortical transcript abundance is causal, but similar effects might be present in other organs and tissues [29] providing insight into molecular mechanisms that contribute to the phenotypic variation in cattle. Subsequently we will discuss some of the genes that were highlighted by the functional analyses and that have been discussed to be involved in the phenotypic manifestation in cattle in other studies.

### Genes involved in reproduction and other traits

*Vimentin* (*VIM*) is a type III intermediate filament that is expressed in cells of mesenchymal origin and has key functions in the formation of the cytoskeletal network, organelle positioning, cell migration and adhesions as well as in cell signaling [32]. *VIM* expression has been shown to be required for the development and establishment of the embryo in cattle. It was reported to be differentially expressed between nuclear transfer and by in vitro fertilization produced embryos [33, 34]. Nowadays in vitro production of embryos is common and approximately 15 % of all embryos are produced by in vitro technologies in cattle [35, 36].

The *luteinizing hormone/chorigonadotropin receptor* (*LHCGR*) is a G-protein-coupled receptor that is mainly known for binding luteinizing hormone and chorionic gonadotropin and mediating their cellular actions. In humans inactivating mutations in the *LHCGR* gene have been shown to cause infertility in both male and female [37]. In addition, *LHCGR* is discussed to be involved in the manifestation of the pregnancy induced Cushing’s Syndrome due to adrenocortical *LHCGR* overexpression that may lead to adrenocortical hyperfunction [38–40]. In cattle, polymorphisms within the *LHCGR* gene have been shown to be associated with calving interval, days to first service and milk yield [41] as well as with superovulation traits [42–44] that are important for generating a high number of eggs for the production of embryos [35, 36].

The *cytochrome P450, family 27, subfamily A, polypeptide 1* (*Cyp27A1*) is a key-enzyme in Vitamin D3 metabolism [45] and is required for bile acid synthesis [46, 47]. In cattle, Vitamin D3 supplementation has been shown to affect dry-matter intake and beef tenderness [48]. In a previous study in the SEGFAM population, metabolites that belong to the sub class “Vitamine D3 and derivates” of Sterol Lipids were important for the classification of temperament types based on prefrontal cortex and serum metabolites [25]. In addition, likewise to *LHCGR*, *Cyp27A1* has also been discussed to be important for reproduction [49] but has also been shown to affect milk yield and somatic cell score [50], an important production and an important health trait in dairy cattle, respectively.

### Genes involved in milk production and other traits

The *xanthine dehydrogenase* (*XDH*) is a xanthine oxidoreductase that belongs to the group of molybdenum iron-sulfur flavin hydroxylases. *XDH* contributes to the detoxification of endogenous or xenobiotic compounds and is involved in the oxidative metabolism of purines [51, 52]. In cattle *XDH* has been shown to be upregulated during the lactation cycle due to its contribution to lipid droplet formation [53]. In addition, *XDH* protein abundance has been shown to increase in the first nine days of lactation in milk serum [54], and [55] suggested *XDH* to be a candidate gene for milk production and mastitis susceptibility based on studies in mouse and cattle.

*Transferrin* (*TF*) together with ferritin is important for acquisition, transport and storage of iron which is essential for many metabolic processes like the synthesis of hemoglobin for oxygen transport [56]. In cattle, *TF* has been shown to be highly polymorphic [57] and a study in Chinese native cattle indicated that *TF* polymorphisms are associated with protein yield, 305-day milk yield and mastitis susceptibility. In addition, they showed that *TF* mRNA expression was higher in mastitis affected in comparison with unaffected mammary tissue [58].

### Genes potentially affecting behavior and temperament

The *aldehyde dehydrogenase 5 family, member A1* (*ALDH5A1*) is a semialdehyde dehydrogenase that is important for the metabolism of γ-aminobutyric acid (GABA) the major inhibitory neurotransmitter in the brain [59]. A differential expression of *ALDH5A1* has been reported in Angus cattle selected for high or low residual feed intake [60]. Increased levels of 2,4-dihidroxy-butanoic acid have been observed in the urine of patients suffering from semialdehyde dehydrogenase deficiency [61] and an accumulation of gamma-hydroxybutyric acid (GHB) and GABA in the central nervous system was reported in ALDH5A1−/− mice [62]. 2,4-dihidroxy-butanoic was priviously identified to be a potential biomarker in Alzheimer’s disease [63] and a temperament type related abundance of 2,4-dihidroxy-butanoic acid as well as of γ-aminobutyric acid acid has also been observed in temperament type specific metabolite profiles of the prefrontal cortex of animals with extrem temperament types deriving from the SEGFAM population [25]. Interestingly *ALDH5A1* is located on chromosome 23 in the genomic region were bovine MHC genes are located. In this genomic region we identified a high number of SNPs that were not only associated with the expression of *ALDH5A1* but also with the expression of some MHC genes like the *MHC class I heavy chain BOLA* and the *major histocompatibility complex BOLA-DQA1*. This genomic region has been reported to be associated with the antibody mediated immune response in Canadian Holstein cows [64].

Another study analyzed the impact of genetic variants on behaviour characteristics assessed in an open field and novel object test within the SEGFAM population [28]. We identified three SNPs (ARS-BFGL-NGS-2942/rs110027993, ARS-BFGL-NGS-98658/rs109674592, ARS-BFGL-NGS-27299/rs109313646) that affect the expression of *NME6* and *RAB32* in this study. The same SNPs were previously identified to be associated with the inactivity in an open field test and the explorative behaviour in a novel object test, respectively [28].

The *NME/NM23 nucleoside diphosphate kinase 6* (*NME6*) is a nucleoside diphosphate kinase. Little is known about its function besides that it is assumed to play a role in cell growth and cell cycle progression [65, 66]. In addition, a recent study in pig indicated that *NME6* is located in a QTL region associated with back fat [67].

*RAB32, member RAS oncogene family* (*RAB32*) belongs to the RAB family of small GTP-binding proteins. It is involved in the biogenesis of lysosome-related organelles like melanosomes [68]. Similar to *NME6* we could not find any additional information other than a suggested role in lipid metabolism [69].

### Ingenuity® upstream analysis

The upstream analysis revealed *PRL*, *ESRRG* and *GNRH1* as upstream or master regulators, providing insight into regulatory mechanisms that are potentially affected by eQTL.

*Prolactin* (*PRL*) is an anterior pituitary protein hormone that is not only important for reproduction but is also involved in the control of behaviours [70]. In cattle especially the effects of *PRL* in regard to lactation and milk production have been investigated. In the last years several studies in cattle investigated the inhibition of PRL-release on milk yield and showed that the inhibition was associated with a decrease in milk yield additionally indicating an important role of *PRL* in the maintenance of lactation [71–73]. The SEGFAM population showed remarkable differences in their milk production [24].

*Gonadotropin-releasing hormone 1* (*GNRH1*) encodes a protein that is secreted and cleaved to form the luteinizing hormone-releasing hormone and the prolactin release-inhibiting factor that regulate *LH* and *FSH* gene expression [74]. *LH* and *FSH* both have been early recognized to be involved in the onset of puberty in cattle and today Gonadotropin-releasing hormone is used to synchronize cows for timed artificial insemination especially in beef cattle [75, 76]. In addition, GnRH signaling was previously identified as a key modifier of differential growth at the onset of puberty in the SEGFAM population [4].

The *Estrogen-Related Receptor Gamma* (*ESRRG*) that belongs to the estrogen receptor-related receptor family has recently been shown to be important in the fibrate-mediated regulation of lipid metabolism genes in a human ApoA-I transgenic mice model [77]. Interestingly *ESRRG* was also identified to be associated with the onset of puberty in cattle [78].

## Conclusions

In the present study we investigated the genetic background of adrenocortical gene expression and identified eQTL affecting the expression levels of genes that have been reported to influence economically important traits in cattle. In addition, some of the genes and molecular pathways affected by eQTL were previously shown to contribute to the phenotypic variation of behaviour, temperament and growth at the onset of puberty in the SEGFAM population. Therefore, eQTL analysis appears to be a useful approach to gain insight into the molecular and genetic background of complex traits in cattle. Additional studies are needed to identify the causal polymorphisms and underlying molecular mechanisms (cis or trans) and the effects of genetic variation on gene expression in other tissues.

## Methods

### Animals and tissue samples

This study comprised 145 F2 cows from the experimental resource cross population (SEGFAM) that was initiated at the Leibniz Institute for Farm Animal Biology (FBN) in the P0 generation by crossing five purebred Charolais sires to purebred German Holstein cows [22]. The population was established by multiple ovulation and embryo transfer, which resulted in five half-sib and full-sib families from which 49, 35, 52, 8 and 1 cows were included in this study, respectively. The cows from the fourth and fifth family were maternally related to animals in the first and third family, respectively. All animals were housed in a loose housing barn under identical environmental and feeding conditions at the Leibniz Institute for Farm Animal Biology (FBN) in Dummerstorf, Germany [24]. The animals were slaughtered at day 30 after parturition in their second lactation following a standardized protocol. Besides other tissues mammary gland tissue and the adrenal gland were immediately taken after slaughter for DNA and RNA extraction, respectively. The adrenal gland was further dissected to separate the adrenal cortex from the medulla. All tissue samples were cut in small pieces, snap-frozen in liquid nitrogen and stored at −80 °C or in liquid nitrogen.

### DNA extraction and SNP genotyping

SNP genotyping was accomplished using Illumina® BovineSNP50 Beadchip v1.0 and v2.0. Genotype calling was performed with the Genotyping Module of the GenomeStudio V2011.1 Software (Illumina®). For this purpose genomic DNA was extracted from mammary gland tissue with the QIAmp DNA Mini Kit (QIAGEN, Hilden, Germany) and prepared for hybridization on the Illumina® BovineSNP50 Beadchip following manufacturer’s instructions. Genotype calling and quality control was performed according to [79] for v1.0 and v2.0 data separately. After quality control and filtering the v1.0 and v2.0 datasets were merged and all samples and SNPs with more than 10 % missing genotypes and a minor allele frequency of less than 5 % were excluded from further analyses. To identify inconsistencies between recorded genotypes and pedigree information, the software PedCheck [80] and a larger dataset including genotypes of P0 sires and all (male and female) F1 and F2 animals was used [81]. The final dataset comprised 37,204 SNPs of 145 animals. Chromosomal positions of SNPs are taken from [82] and are based on genome assembly UMD3.1.

### RNA extraction and microarray hybridization

Total RNA was isolated from the adrenal cortex of 145 animals using TRI Reagent (Sigma, Taufkirchen, Germany). After DNase I treatment the RNA was further purified with the RNeasy Kit (QIAGEN, Hilden, Germany) following the manufacturers recommendations. RNA was quantified using the NanoDrop ND-1000 spectrophotometer (Peqlab, Erlangen, Germany). Integrity of RNA was checked by running 1 μg of RNA on a 1 % agarose gel. Absence of DNA contamination was verified by PCR amplifying fragments of GAPDH with RNA as a template. For hybridization, 500 ng of total RNA were amplified using Ambion WT Expression Kit (Affymetrix). Subsequently, 5.5 μg of the resulting cDNA was fragmented and labeled using the Affymetrix Terminal Labeling Kit. The fragmented cDNA was hybridized to the microarray using the Affymetrix Hyb-WashStain Kit and Affymetrix standard protocols. Fluidic station protocol was FS450_0001.

For expression profiling the custom GeneChip Bovine Gene v1 Array was used. Affymetrix designed the GeneChip Bovine Gene v1 Array based on Ensemble and RefSeq predictions for the Genome Bos Taurus Build 4.0. The design was targeted to develop a whole Genome Expression Array with approximately 25 probes per transcript distributed over the whole length of each transcript. In total 194,712 probe sets targeting at approximately 24,000 transcripts are implemented on the array. In addition standard Affymetrix controls for hybridization and labeling efficiency as well as for non-specific binding are included on the array. Gene-level analysis was performed using Affymetrix® Expression Console™ Software. Microarray raw data were preprocessed using the RMA algorithm [83]. Quality control was performed in accordance to [84]. In addition, the detection above background algorithm was used to identify expressed genes. Probe sets that were present in 75 % of all samples and transcripts with at least 50 % of all probe sets present were included in the analyses. The final dataset comprised expression values for 10,986 transcripts of 145 animals. For annotation the NetAffx [85] release 34 chromosomal positions of the transcripts were used that are based on genome assembly UMD3.1. In addition the Ingenuity® gene annotation is provided including information about transcript cluster ids that target the same gene. The microarray data are deposited at Gene Expression Omnibus database [86] (GEO: GSE75371).

### Data pre-processing

After quality control and filtering the expression data was further pre-processed to account for systemic effects. Furthermore, the statistical test used for eQTL analysis required the data to be decorrelated. Since we expected gametic phase disequilibrium for some of the loci, we refrained from estimating kinship from SNP data but preferred the use of kinship estimates from pedigree information. Therefore the following sire-dam model was applied in ASReml [87] to account for fixed effects of season and year and age at slaughter as well as for relatedness:

$$ {y}_i={s}_j+\beta {x}_i + \mathrm{\frac{1}{2}}\ \left({a}_{if}+{a}_{im}\right)\kern0.5em +\kern0.5em {\varepsilon}_i $$

where *y*_*i*_ is the expression value for transcript *y* of animal *i* (*i* = 1,…, 145), *s*_*j*_ is the fixed effect of year and season (*j* = 1,…, 25) at slaughter, *x*_*i*_ is the age in days of animal *i* at slaughter, *a*_*if*_ is the additive-genetic effect of the sire of animal *i*, *a*_*im*_ is the additive-genetic effect of the dam of animal *i* and *ε*_*i*_ is the random residual effect. To estimate the additive-genetic effects the complete pedigree data comprising 38 P0 and 52 F1 animals was used. The residuals comprise the Mendelian sampling effect as an uncorrelated genetic component as well as a random deviation. For further analysis the residuals were considered as de-correlated expression data.

### eQTL analysis

For eQTL analysis an adaptive linear rank test [27] was applied. The test was optimized to meet the challenge of non-normally distributed expression data in eQTL studies. This two stage procedure first calculates selector statistics for skewness and tail length of the de-correlated expression data. Then an appropriate linear rank test depending on the selector statistics is applied to identify differences in the expression levels per genotype group. Thus, for each pair of SNP and transcript a linear rank test is selected that is supposed to have large statistical power for the respective actual distribution. The available tests are Kruskal-Wallis test [88], median test [89], long tails test [90], short tails test [91], right skewness and left skewness test [92]. To account for multiple testing we applied a global significance level of 0.1 for Benjamini-Hochberg adjusted *p*-values [93] (considering all 374 million performed tests), which is approximately equivalent to a significance level of 1.3x10^−7^ for unadjusted *p*-values. This global false discovery rate (FDR) threshold of 0.1 is less conservative for trans associations than usual, but it is very conservative for cis associations, since it corresponds to a FDR threshold of approximately 0.0035 for an eQTL analysis in which only cis associations (approximately 13 million) are considered for the calculation of the Benjamini-Hochberg adjusted *p*-values. This was done because the majority of associations was expected between SNPs and transcripts for which the encoding gene of the transcript was located on the same chromosome in close vicinity to the SNP.

### Ingenuity® pathway analysis

The functional and upstream analyses were performed through the use of QIAGEN’s Ingenuity® Pathway Analysis (IPA, QIAGEN Redwood City, www.qiagen.com/ingenuity). The purpose of the functional analysis was to determine if the 129 identified genes are statistically significant over-represented (more genes than expected by chance) for a specific molecular or cellular function in comparison to a reference-set of genes. Similar to the functional analysis, the Ingenuity® upstream analysis was performed to measure whether there is a statistically significant overlap between the 129 identified genes and the genes that are regulated by a specific transcription regulator. For both tests IPA uses a Fisher’s Exact Test to determine the significance of the over-representation or overlap in comparison to a reference set. As reference set for the functional analysis the Ingenuity® Knowledge Base was chosen, because the reference network that is used to identify the upstream and master (upstream and downstream) regulators in the upstream analysis is based on the Ingenuity® Knowledge Base. Reported *p*-values are Benjamini-Hochberg adjusted.

## Additional files

Additional file 1: List of eQTL. A complete list of eQTL comprising the chromosomal position and annotation of the SNPs and transcripts as well as the results of the adaptive linear rank test, the Kruskal Wallis-test and ANOVA are provided including the *p*-values for all tests and the corresponding Benjamini-Hochberg adjusted *p*-values for the adaptive linear rank test. (XLSX 75 kb)
Additional file 2: Linkage disequilibrium decay plot. Genome-wide linkage disequilibrium (LD) decay plot for 145 cows of a F2 resource population deriving from a cross between Charolais and German Holstein founder breeds based on 37,204 polymorphic single nucleotide polymorphism (SNP) markers. The pairwise linkage disequilibrium (r^2^) between all pairs of SNPs on each chromosome was calculated and plotted against the genetic distance of the two markers. (PDF 521 kb)

## Abbreviations

ANOVA: Analysis of variance
cDNA: Complementary DNA
Chr: Chromosome
eQTL: Expression quantitative trait loci
FDR: False discovery rate
GABA: γ-aminobutyric acid
GHB: Gamma-hydroxybutyric acid
MB: Mega base-pairs
PCR: Polymerase chain reaction
QTL: Quantitative trait loci
SNP: Single nucleotide polymorphism

## References

[1] Cazier JB, Kaisaki PJ, Argoud K, Blaise BJ, Veselkov K, Ebbels TMD (2012). Untargeted metabolome quantitative trait locus mapping associates variation in urine glycerate to mutant glycerate kinase. J Proteome Res.

[2] Portelli MA, Siedlinski M, Stewart CE, Postma DS, Nieuwenhuis MA, Vonk JM (2014). Genome-wide protein QTL mapping identifies human plasma kallikrein as a post-translational regulator of serum uPAR levels. FASEB J.

[3] Ponsuksili S, Murani E, Brand B, Schwerin M, Wimmers K (2011). Integrating expression profiling and whole-genome association for dissection of fat traits in a porcine model. J Lipid Res.

[4] Widmann P, Reverter A, Fortes MR, Weikard R, Suhre K, Hammon H (2013). A systems biology approach using metabolomic data reveals genes and pathways interacting to modulate divergent growth in cattle. BMC Genomics.

[5] Yang Y, Bu D, Zhao X, Sun P, Wang J, Zhou L (2013). Proteomic Analysis of Cow, Yak, Buffalo, Goat and Camel milk whey proteins: quantitative differential expression patterns. J Proteome Res.

[6] Kadarmideen HN (2014). Genomics to systems biology in animal and veterinary sciences: progress, lessons and opportunities. Livest Sci.

[7] Hemsworth PH, Coleman GJ, Barnett JL, Borg S (2000). Relationships between human-animal interactions and productivity of commercial dairy cows. J Anim Sci.

[8] Müller R, von Keyserlingk MAG (2006). Consistency of flight speed and its correlation to productivity and to personality in Bos taurus beef cattle. Appl Anim Behav Sci.

[9] Sutherland MA, Rogers AR, Verkerk GA (2012). The effect of temperament and responsiveness towards humans on the behavior, physiology and milk production of multi-parous dairy cows in a familiar and novel milking environment. Physiol Behav.

[10] Burdick N, Randel R, Carroll J, Welsh T (2011). Interactions between temperament, stress, and immune function in cattle. Int. J. Zoonoses.

[11] Burdick NC, Banta JP, Neuendorff DA, White JC, Vann RC, Laurenz JC (2009). Interrelationships among growth, endocrine, immune, and temperament variables in neonatal Brahman calves. J Anim Sci.

[12] Kasimanickam R, Asay M, Schroeder S, Kasimanickam V, Gay JM, Kastelic JP (2014). Calm temperament improves reproductive performance of beef cows. Reprod Domest Anim.

[13] Cooke RF, Arthington JD, Araujo DB, Lamb GC (2009). Effects of acclimation to human interaction on performance, temperament, physiological responses, and pregnancy rates of Brahman-crossbred cows. J Anim Sci.

[14] Nussey S, Whitehead S (2001). The adrenal gland. Endocrinology: an integrated approach.

[15] Boscaro M, Barzon L, Fallo F, Sonino N (2001). Cushing's syndrome. Lancet.

[16] Piaditis G, Markou A, Papanastasiou L, Androulakis II, Kaltsas G (2015). Progress in aldosteronism: a review of the prevalence of primary aldosteronism in pre-hypertension and hypertension. Eur J Endocrinol.

[17] Ten S, New M, Maclaren N (2001). Addison`s Disease 2001. J Clin Endocrinol Metab.

[18] Wagner WC, Oxenreider SL (1972). Adrenal function in the cow. Diurnal changes and the effects of lactation and neurohypophyseal hormones. J Anim Sci.

[19] Wagner WC, Hansel W (1969). Reproductive physiology of the post partum cow. J Reprod Fertil.

[20] Poleti MD, DeRijk RH, Rosa AF, Moncau CT, Oliveira PS, Coutinho LL (2015). Genetic variants in glucocorticoid and mineralocorticoid receptors are associated with concentrations of plasma cortisol, muscle glycogen content, and meat quality traits in male Nellore cattle. Domest Anim Endocrinol.

[21] Reyer H, Ponsuksili S, Wimmers K, Murani E (2014). Association of N-terminal domain polymorphisms of the porcine glucocorticoid receptor with carcass composition and meat quality traits. Anim Genet.

[22] Kühn C, Bellmann O, Voigt J, Wegner J, Guiard V, Ender K (2002). An experimental approach for studying the genetic and physiological background of nutrient transformation in cattle with respect to nutrient secretion and accretion type. Arch Tierz.

[23] Weikard R, Altmaier E, Suhre K, Weinberger K, Hammon H, Albrecht E (2010). Metabolomic profiles indicate distinct physiological pathways affected by two loci with major divergent effect on Bos taurus growth and lipid deposition. Physiol Genomics.

[24] Hammon HM, Metges CC, Schulz A, Junghans P, Steinhoff J, Schneider F (2010). Differences in milk production, glucose metabolism, and carcass composition of 2 Charolais X Holstein F2 families derived from reciprocal paternal and maternal grandsire crosses. J Dairy Sci.

[25] Brand B, Hadlich F, Brandt B, Schauer N, Graunke KL, Langbein J (2015). Temperament type specific metabolite profiles of the prefrontal cortex and serum in cattle. PLoS One.

[26] Graunke LK, Nürnberg G, Repsilber D, Puppe B, Langbein J (2013). Describing temperament in an ungulate: a multidimensional approach. PLoS One.

[27] Szymczak S, Scheinhardt MO, Zeller T, Wild PS, Blankenberg S, Ziegler A (2013). Adaptive linear rank tests for eQTL studies. Stat Med.

[28] Friedrich J, Brand B, Ponsuksili S, Graunke KL, Langbein J, Knaust J (2015). Detection of genetic variants affecting cattle behaviour and their impact on milk production: a genome-wide association study. Anim Genet.

[29] Rockman MV, Kruglyak L (2006). Genetics of global gene expression. Nat Rev Genet.

[30] Williams RBH, Chan EKF, Cowley MJ, Little PFR (2007). The influence of genetic variation on gene expression. Genome Res.

[31] Holloway B, Luck S, Beatty M, Rafalski JA, Li B (2011). Genome-wide expression quantitative trait loci (eQTL) analysis in maize. BMC Genomics.

[32] Ivaska J, Pallari HM, Nevo J, Eriksson JE (2007). Novel functions of vimentin in cell adhesion, migration, and signaling. Exp Cell Res.

[33] Pfister-Genskow M, Myers C, Childs LA, Lacson JC, Patterson T, Betthauser JM (2005). Identification of differentially expressed genes in individual bovine preimplantation embryos produced by nuclear transfer: improper reprogramming of genes required for development. Biol Reprod.

[34] Maddox-Hyttel P, Alexopoulos NI, Vajta G, Lewis I, Rogers P, Cann L (2003). Immunohistochemical and ultrastructural characterization of the initial post-hatching development of bovine embryos. Reproduction.

[35] Mapletoft RJ, Hasler JF (2005). Assisted reproductive technologies in cattle: a review. Rev Sci Tech.

[36] Rodriguez-Martinez H (2012). Assisted reproductive techniques for cattle breeding in developing countries: a critical appraisal of their value and limitations. Reprod Domest Anim.

[37] Layman LC (2002). Human gene mutations causing infertility. J Med Genet.

[38] Chui MH, Ozbey NC, Ezzat S, Kapran Y, Erbil Y, Asa S (2009). Case report: Adrenal LH/hCG receptor overexpression and gene amplification causing pregnancy-induced Cushing's syndrome. Endocr Pathol.

[39] Bernichtein S, Alevizaki M, Huhtaniemi I (2008). Is the adrenal cortex a target for gonadotropins?. Trends Endocrinol Metab.

[40] Carlson HE (2007). Human adrenal cortex hyperfunction due to LH/hCG. Mol Cell Endocrinol.

[41] Hastings N, Donn S, Derecka K, Flint AP, Woolliams JA (2006). Polymorphisms within the coding region of the bovine luteinizing hormone receptor gene and their association with fertility traits. Anim Genet.

[42] Yang WC, Tang KQ, Li SJ, Chao LM, Yang LG (2012). Polymorphisms of the bovine luteinizing hormone/choriogonadotropin receptor (LHCGR) gene and its association with superovulation traits. Mol Biol Rep.

[43] Luo W, Gumen A, Haughian JM, Wiltbank MC (2011). The role of luteinizing hormone in regulating gene expression during selection of a dominant follicle in cattle. Biol Reprod.

[44] Yu Y, Pang Y, Zhao H, Xu X, Wu Z, An L (2012). Association of a missense mutation in the luteinizing hormone/choriogonadotropin receptor gene (LHCGR) with superovulation traits in Chinese Holstein heifers. J Anim Sci Biotechnol.

[45] Sawada N, Sakaki T, Ohta M, Inouye K (2000). Metabolism of vitamin D3 by human CYP27A1. Biochem Biophys Res Commun.

[46] Goodwin B, Gauthier KC, Umetani M, Watson MA, Lochansky MI, Jon LC (2003). Identification of bile acid precursors as endogenous ligands for the nuclear xenobiotic pregnane X receptor. Proc Natl Acad Sci U S A.

[47] Chiang JYL (2009). Bile acids: regulation of synthesis. J Lipid Res.

[48] Karges K, Brooks JC, Gill DR, Breazile JE, Owens FN, Morgan JB (2001). Effects of supplemental vitamin D3 on feed intake, carcass characteristics, tenderness, and muscle properties of beef steers. J Anim Sci.

[49] Bauersachs S, Ulbrich SE, Zakhartchenko V, Minten M, Reichenbach M, Reichenbach HD (2009). The endometrium responds differently to cloned versus fertilized embryos. Proc Natl Acad Sci U S A.

[50] Pimentel ECG, Bauersachs S, Tietze M, Simianer H, Tetens J, Thaller G (2011). Exploration of relationships between production and fertility traits in dairy cattle via association studies of SNPs within candidate genes derived by expression profiling. Anim Genet.

[51] Xu P, Huecksteadt TP, Hoidal JR (1996). Molecular cloning and characterization of the human xanthine dehydrogenase gene (XDH). Genomics.

[52] Weseler AR, Bast A (2010). Oxidative stress and vascular function: implications for pharmacologic treatments. Curr Hypertens Rep.

[53] Bionaz M, Loor J (2008). Gene networks driving bovine milk fat synthesis during the lactation cycle. BMC Genomics.

[54] Zhang L, Boeren S, Hageman JA, van Hooijdonk T, Vervoort J, Hettinga K (2015). Bovine milk proteome in the first 9 days: protein interactions in maturation of the immune and digestive system of the newborn. PLoS One.

[55] Ogorevc J, Kunej T, Razpet A, Dovc P (2009). Database of cattle candidate genes and genetic markers for milk production and mastitis. Anim Genet.

[56] Ponka P, Beaumont C, Richardson DR (1998). Function and regulation of transferrin and ferritin. Semin Hematol.

[57] Lukac D, Vidovic V, Nemeš Z, Stupar M, Popovic-Vranješ A (2013). Genotypic frequencies of the ß-lactoglobulin, k-casein and transferrin in Serbian Holstein-Friesian dairy cattle. Mljekarstvo/Dairy.

[58] Ju Z, Li Q, Huang J, Hou M, Li R, Li J (2011). Three novel SNPs of the bovine Tf gene in Chinese native cattle and their associations with milk production traits. Genet Mol Res.

[59] McCormick DA (1989). GABA as an inhibitory neurotransmitter in human cerebral cortex. J Neurophysiol.

[60] Chen Y, Gondro C, Quinn K, Herd RM, Parnell PF, Vanselow B (2011). Global gene expression profiling reveals genes expressed differentially in cattle with high and low residual feed intake. Anim Genet.

[61] Brown GK, Cromby CH, Manning NJ, Pollitt RJ (1987). Urinary organic acids in succinic semialdehyde dehydrogenase deficiency: evidence of a-oxidation of 4-hydroxybutyric acid, interaction of succinic semialdehyde with pyruvate dehydrogenase and possible secondary inhibition of mitochondrial ß-oxidation. J Inherit Metab Dis.

[62] Knerr I, Pearl PL, Bottiglieri T, Carter Snead O, Jakobs C, Gibson KM (2007). Therapeutic concepts in succinate semialdehyde dehydrogenase (SSADH; ALDH5a1) deficiency (gamma-hydroxybutyric aciduria). Hypotheses evolved from 25 years of patient evaluation, studies in Aldh5a1**−/−** mice and characterization of gamma-hydroxybutyric acid pharmacology. J Inherit Metab Dis.

[63] Oresic M, Hyotylainen T, Herukka SK, Sysi-Aho M, Mattila I, Seppanan-Laakso T (2011). Metabolome in progression to Alzheimer's disease. Transl Psychiatry.

[64] Thompson-Crispi K, Sargolzaei M, Ventura R, Abo-Ismail M, Miglior F, Schenkel F (2014). A genome-wide association study of immune response traits in Canadian Holstein cattle. BMC Genomics.

[65] Tsuiki H, Nitta M, Furuya A, Hanai N, Fujiwara T, Inagaki M (2000). A novel human nucleoside diphosphate (NDP) kinase, Nm23-H6, localizes in mitochondria and affects cytokinesis. J Cell Biochem.

[66] Desvignes T, Pontarotti P, Fauvel C, Bobe J (2009). Nme protein family evolutionary history, a vertebrate perspective. BMC Evol Biol.

[67] Onteru SK, Gorbach DM, Young JM, Garrick DJ, Dekkers JCM, Rothschild MF (2013). Whole genome association studies of residual feed intake and related traits in the pig. PLoS One.

[68] Bultema JJ, Boyle JA, Malenke PB, Martin FE, Dell'Angelica EC, Cheney RE (2014). Myosin vc interacts with Rab32 and Rab38 proteins and works in the biogenesis and secretion of melanosomes. J Biol Chem.

[69] Wang C, Liu Z, Huang X (2012). Rab32 is important for autophagy and lipid storage in Drosophila. PLoS One.

[70] Freeman ME, Kanyicska B, Lerant A, Nagy G (2000). Prolactin: structure, function, and regulation of secretion. Physiol Rev.

[71] Lacasse P, Lollivier V, Bruckmaier RM, Boisclair YR, Wagner GF, Boutinaud M (2011). Effect of the prolactin-release inhibitor quinagolide on lactating dairy cows. J Dairy Sci.

[72] Boutinaud M, Lollivier V, Finot L, Bruckmaier RM, Lacasse P (2012). Mammary cell activity and turnover in dairy cows treated with the prolactin-release inhibitor quinagolide and milked once daily. J Dairy Sci.

[73] Ollier S, Zhao X, Lacasse P (2015). Effects of feed restriction and prolactin-release inhibition at drying-off on susceptibility to new intramammary infection in cows. J Dairy Sci.

[74] Thompson IR, Kaiser UB (2014). GnRH pulse frequency-dependent differential regulation of LH and FSH gene expression. Mol Cell Endocrinol.

[75] Schams D, Schallenberger E, Gombe S, Karg H (1980). Endocrine patterns associated with puberty in male and female cattle. J Reprod Fertil Suppl.

[76] Day ML (2015). State of the art of GnRH-based timed AI in beef cattle. Anim Reprod.

[77] Sanoudou D, Duka A, Drosatos K, Hayes KC, Zannis VI (2010). Role of Esrrg in the fibrate-mediated regulation of lipid metabolism genes in human ApoA-I transgenic mice. Pharmacogenomics J.

[78] Fortes MRS, Reverter A, Zhang Y, Collis E, Nagaraj SH, Jonsson NN (2010). Association weight matrix for the genetic dissection of puberty in beef cattle. Proc Natl Acad Sci U S A.

[79] Illumina, Infinium Genotyping Data Analysis. http://www.illumina.com/Documents/products/technotes/technote_infinium_genotyping_data_analysis.pdf. Accessed 1 Oct 2016.

[80] O'Connell JR, Weeks DE (1998). PedCheck: a program for identification of genotype incompatibilities in linkage analysis. Am J Hum Genet.

[81] Knaust J, Hadlich F, Weikard R, Kuehn C (2016). Epistatic interactions between at least three loci determine the “rat-tail” phenotype in cattle. Genet Sel Evol.

[82] Fadista J, Bendixen C (2012). Genomic position mapping discrepancies of commercial SNP chips. PLoS One.

[83] Irizarry RA, Bolstad BM, Collin F, Cope LM, Hobbs B, Speed TP (2003). Summaries of Affymetrix GeneChip probe level data. Nucl Acids Res.

[84] Affymetrix, Quality Assessment of Exon and Gene Arrays. http://media.affymetrix.com/support/technical/whitepapers/exon_gene_arrays_qa_whitepaper.pdf. Accessed 1 Oct 2016.

[85] Liu G, Loraine AE, Shigeta R, Cline M, Cheng J, Valmeekam V (2003). NetAffx: Affymetrix probesets and annotations. Nucl Acids Res.

[86] Gene Expression Omnibus (GEO). http://www.ncbi.nlm.nih.gov/projects/geo/. Accessed 1 Oct 2016.

[87] Gilmour AR, Gogel BJ, Cullis BR, Thompson R (2006). ASReml User Guide Release 2.0. VSN International Ltd, Hemel Hempstead, HP1 1ES, UK.

[88] Kruskal WH (1952). A nonparametric test for the several sample problem. Ann. Math. Stat..

[89] Mood AM, Graybill FA, Boes DC (1974). Introduction to the Theory of Statistics.

[90] Büning H (1997). Robust analysis of variance. J Appl Stat.

[91] Gastwirth JL (1965). Percentile modifications of two sample rank tests. J Am Stat Assoc.

[92] Hogg RV, Fisher DM, Randles RH (1975). A two sample adaptive distribution-free test. J Am Stat Assoc.

[93] Benjamini Y, Hochberg Y (1995). Controlling the False Discovery Rate - A practical and powerful approach to multiple testing. J R Stat Soc Series B Stat Methodol.

