# Erratum to: Adrenal cortex expression quantitative trait loci in a German Holstein × Charolais cross

**DOI:** 10.1186/s12863-016-0450-x

**Published:** 2016-11-30

**Authors:** Bodo Brand, Markus O. Scheinhardt, Juliane Friedrich, Daisy Zimmer, Norbert Reinsch, Siriluck Ponsuksili, Manfred Schwerin, Andreas Ziegler

**Affiliations:** 1 Institute for Genome Biology, Leibniz Institute for Farm Animal Biology, Wilhelm-Stahl-Allee, Dummerstorf, Germany; 2 Current affiliation: Institute for Farm Animal Research and Technology, University of Rostock, Justus-von-Liebig-Weg, 18059 Rostock, Germany; 3 Institute of Medical Biometry and Statistics, University of Lübeck, Ratzeburger Allee, Lübeck, Germany; 4 Institute for Farm Animal Research and Technology, University of Rostock, Justus-von-Liebig-Weg, Rostock, Germany; 5 Institute for Genetics and Biometry, Leibniz Institute for Farm Animal Biology, Wilhelm-Stahl-Allee, Dummerstorf, Germany; 6 Center for Clinical Trials, University of Lübeck, Ratzeburger Allee, Lübeck, Germany; 7 School of Mathematics, Statistics and Computer Science, University of KwaZulu-Natal, Pietermaritzburg, South Africa

## Erratum

Unfortunately, after publication of this article [1], it was noticed that an error in the PDF to an equation was introduced during the production process. On page 8 of the PDF, the equation reads:

$$ {y}_i={s}_j+\beta {x}_i+\S \Big|\left({a}_{if}+{a}_{im}\right)+{\varepsilon}_i $$

The equation should have read:

$$ {y}_i={s}_j+\beta {x}_i+\frac{1}{2}\ \left({a}_{if}+{a}_{im}\right)+{e}_i $$

The original article has been updated to correct this error.
