# Supplementary figures and images for: Adrenal cortex expression quantitative trait loci in a German Holstein × Charolais cross
Source: BMC Genet. 2016 Oct 6;17:135. doi: 10.1186/s12863-016-0442-x (PMC5053117; doi:10.1186/s12863-016-0442-x)

Linkage disequilibrium ( $r^2$ )

1.00  
0.75  
0.50  
0.25

0

50

100

150

Distance (MB)

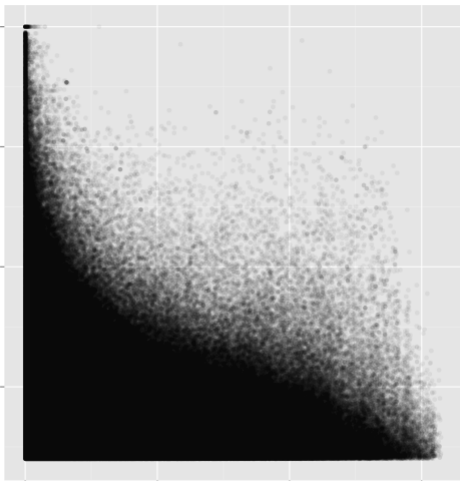

Supplement: Additional file 2: — Linkage disequilibrium decay plot. Genome-wide linkage disequilibrium (LD) decay plot for 145 cows of a F2 resource population deriving from a cross between Charolais and German Holstein founder breeds based on 37,204 polymorphic single nucleotide polymorphism (SNP) markers. The pairwise linkage disequilibrium (r2) between all pairs of SNPs on each chromosome was calculated and plotted against the genetic distance of the two markers. (PDF 521 kb) [file 12863_2016_442_MOESM2_ESM.pdf]
